# COVID-19 and community pharmacy services in Pakistan: challenges, barriers and solution for progress

**DOI:** 10.1186/s40545-020-00240-4

**Published:** 2020-06-15

**Authors:** Muhammad Atif, Iram Malik

**Affiliations:** Department of Pharmacy, The Islamia University of Bahawalpur, Bahawalpur, Punjab Pakistan

**Keywords:** Drug Regulatory Authority of Pakistan, DRAP, Pharm-D, Punjab Drug Rules 2007, Drugs Act 1976, Pharmacy proprietors, Pharmacist

## Abstract

In the wake of atrocious rise in COVID-19 cases, developed countries are leveraging a range of community pharmacy services with the goal of improving access to essential medication and healthcare services. While in the developing nations, including Pakistan, pharmacists are unable to perform COVID-19 containment roles in community, since presence of pharmacists at community pharmacy settings and delivery of pharmacy services have historically been plagued by shortcomings at various levels. In this document, we identified these shortcomings which need to be resolved on many fronts. Broadly, a number of intertwined government related, public related, academic curricula and pharmacist related, and drug retailers’ related factors refrain community pharmacists from performing and facilitating Pakistan’s fragile public and healthcare system in the midst of COVID-19 pandemic. Government led multifaceted approaches are urgently needed to strengthen this unrecognized domain and thereby effectively combat COVID-19 by utilizing community pharmacy services, as evidenced in the developed world. [Note: Part of this article is published in Pakistan Observer Newspaper; dated 17 May 2020).

## Main text

COVID-19 has put unprecedented pressure on healthcare systems of every nation [1]. Specifically, owing to a number of voids in their healthcare system, the developing nations are unable to meet the healthcare needs of the population and adopt the recommended COVID-19 response strategies to flatten the contagion curve. To this end, there is emerging recognition across the globe that community pharmacists are in a unique position to provide medicines, therapeutics, vaccines and essential healthcare services in the wake of atrocious rise in COVID-19 cases [2]. Many countries across the globe are leveraging a range of community pharmacy services with the goal of improving access to essential medication and healthcare services. As a result, community pharmacists in developed nations, such as China, Australia, Canada, the United States (US) and the United Kingdom (UK) have acted swiftly and are offering a broad range of the quality pharmaceutical service [3–7]. These include, drug review and monitoring, clinical consultation and treatment, medication therapy management, pertinent drug information, assessment of patients and their treatment outcomes, and mitigation of drug shortages through various strategies etc. These evidences from developed countries provides insight to the resource-deprived nations – with fragmented health care systems – for planning and operating pharmacy services to combat current and future epidemics.

In Pakistan, community pharmacy services are highly warranted as the devastating COVID-19 pandemic is swiftly spreading and the country does not have the capacity to meet the international standards of care due to a number of public health and healthcare system related vulnerabilities. Despite being described under the Drug Regulatory Authority of Pakistan (DRAP) Act 2012, a majority of pharmacy services don’t exist in the country and only a few core services [8], such as dispensing, storage, distribution and management of therapeutic goods are available at drug retail outlets. However, there is a mounting list of previously known threats to the optimal use of medicines (e.g., adverse drug reactions, medication errors, misuse of controlled substances and excessive) [9] and medicine-related practices (e.g., inappropriate use of medicines, especially antibiotics, formulation issues, over-the-counter (OTC) availability of prescription medicines, unsafe storage and disposal of medicines, and poor availability of medicines) that demand advanced community pharmacy services in the patient’s welfare [10–15]. Whilst, in the current situation, when healthcare system is buckled under the burden of public health crisis and the country is attempting to adhere to the recommended preventive measures against COVID-19, full-blown community pharmacy services are urgently needed. The importance of community pharmacy services during COVID-19 havoc could be gauged by the fact that many developed nations have amended pharmaceutical policies, introduced reimbursement schemes and released new instructions for community pharmacies to expand pharmacy services [3–7]. For Pakistan, however, this may not be easy, since both the presence of pharmacist and provision of patient centered pharmacy services at community settings have historically been plagued by shortcomings at various levels. Therefore, pharmacists are currently not able to perform COVID-19 control roles in public. This commentary highlights a set of factors that refrain community pharmacists from performing and facilitating Pakistan’s fragile public health and healthcare system in the midst of COVID-19.

### Government related factors

In response to ongoing public health crisis, regulatory authorities throughout the world have been found to play a key role in ensuring the delivery of essential patient care services through utilization of community pharmacy services [3–7]. However, the community pharmacy services are inadequately provided in Pakistan mainly due to lack of attention at government level [8]. Healthcare regulators in Pakistan regulate community pharmacies as ordinary commercial entities rather than considering these as healthcare settings and pharmacists as promising healthcare professionals. Though, rules and regulations in the form of Drugs Act, 1976, Pharmacy Act, 1967 and Punjab Drug Rules 2007 are in place, but partially implemented specifically in terms of assurance of qualified persons’ presence at the community pharmacy settings [16–18]. Recently, Atif and colleagues revealed that there is an excessive breach of existing rules in the country; such as the owners of drug retail outlets do not mandate availability of pharmacist at their premises, and pharmacists rent out their pharmacy license in lieu of small sum of money and this is even without assuring their presence on the premises [8]. Besides regulatory loopholes, the government resists underwriting facilities for community pharmacy proprietors, such as fair profit margins (difference between retail and trade price), low cost electricity and non-profit loans. Analogous to previous patterns, Pakistani healthcare regulators have not yet announced any sort of support to this sector despite the COVID-19 triggered a high need for community pharmacy services.

### Public or patient related factors

The other factors that place the establishment of community pharmacy services at stake are relevant to the general public or patients. The majority of the Pakistani population has low rates of health literacy [19]. As a result, pharmacy customers rarely seek and acknowledge pharmacy services. Moreover, massive proportions of people in Pakistan only recognize physicians as qualified and capable healthcare practitioners [20], while regard pharmacists as purveyor of medicines. This in effect reduces the demand of pharmacists at these settings. In brief, pharmacist’s identity in community pharmacy is questioned. Their abilities are marked unreliable, and are regarded as pre-packaged drug dispensers. However, as a result of reduced public enthusiasm for visiting hospitals due to fear of catching COVID-19, community pharmacists around the globe have a great opportunity to gain recognition as competent healthcare practitioners. The demand of telepharmacy and home delivery of medication and services has grown globally along with other key services. The patients in need of these services are interacting and acknowledging the full potential of community pharmacists. The same could happen in Pakistan if government supports pharmacists and pharmacy proprietor in offering a set of advanced pharmacy services.

### Academic curriculum and pharmacist related factors

Considering the rapid spread of COVID-19 and the limited frontline healthcare personnel and diagnostic facilities, healthcare regulators are looking for strategies to meet the healthcare needs of massive population. There are growing discussions about engagement of community pharmacists in different patient-centered activities. Aside from previously recognized advanced patient care services, community pharmacists are anticipated to be tasked with testing, treatment and immunization of communities. However, aspect that may negatively affects the adoption of these much needed envisaged community pharmacy services in Pakistan is the lack of expertise of pharmacists resulting from deficits in academic curricula and other pharmacist related factors [19, 21]. Although, a transition from the four-years B. Pharm program to the five-year Pharm-D program has been observed in Pakistan [22], the content of pharmacy curriculum does not comply with the needs of advanced pharmacy services [8]. Moreover, clinical sessions and clinical research at under-graduate stage are introduced only as a formality. Pharmacy students, therefore, lack the necessary knowledge and competence regarding advanced pharmacy services [23]. This in turn impacts self-confidence of pharmacists and ultimately the standard of even core pharmacy services, including patient counseling skills [19]. Fresh graduates are also reluctant to acquire professional skills at community pharmacies owing to lack of recognition and low remuneration. In this episode of COVID-19, this attitude of Pakistani pharmacists refrains them from possible interaction with the public and patients.

Furthermore, a large number of pharmacy graduates in Pakistan are female, however, due to socio cultural barriers and traditional orthodox society their presence at the community pharmacies is sparse [24]. Therefore, the low uptake of community pharmacy services in the country during COVID-19 is highly related to this gender imbalance driven low availability of experienced community pharmacists.

### Drug store proprietor related factors

In the wake of COVID-19, nations are facing a huge challenge associated with access to essential medicines. In order to mitigate medication disruption, community pharmacies in the presence of pharmacists have been temporarily authorized to offer additional services, such as medicines compounding, re-packaging of non-prescription medicines, digital image prescriptions and continued dispensing of essential prescription only medicine without a prescription. Nevertheless, authorizations for these services in Pakistan will be curtailed due to unavailability of pharmacists at community settings, which is fuelled by a range of aspects at pharmacy proprietor level. First, fully compliant drug store proprietors are earning less owing to low profit margin in medicines and related products. As a result, they cannot fulfill even reasonable salary demands of pharmacists, which is one of the key factors why pharmacists in Pakistan are unwilling to opt this profession as a full-time job. Second, the proprietors tend to sale “prescription only” and “controlled drugs” easily in the absence of a qualified pharmacist. Therefore, most of pharmacy owners infringe the regulation and avoid recruiting pharmacists at their premises. Third, proprietors do not want to pay fresh pharmacists merely because of their qualification. This is because fresh pharmacists have minimal competency to fill the prescriptions and counsel patients. On the other hand, pharmacists have low demand from the pharmacy customers. Pharmacists on the other hand find it humiliating to work as a sale staff (i.e., prescription filling without patient counseling) after studying a nerve racking five-year professional course. Given the foregoing reasons, it is very difficult not only for community pharmacists to integrate themselves in the community pharmacy settings during COVID-19, but also for pharmacy owners to satisfy the expectations of pharmacists.

### Concluding remarks

There are a number of intertwined government, public, academia, curriculum, pharmacist and drug retailers related factors in Pakistan which are hindering the establishment of community pharmacy services regardless of COVID-19 driven high demand for such services. Government led multifaceted approaches are urgently needed to strengthen this unrecognized domain. The existence of a multi-stakeholder committee led by pharmaceutical regulators and foreign experts appears to be essential in tackling these challenges. The government level initiatives are urgently required to attract and encourage pharmacists to perform their role in community pharmacies during ongoing pandemic.

The government should consider the provision of sufficient interest free loans to the pharmacy graduates which will enable them to open their own pharmacies. On the other hand, the government should urgently consider increasing profit margins for the proprietors (i.e., increase in the difference between trade and sale price) and later emphasizing them to ensure the presence of pharmacists at their setting. The Pharmacy Council of Pakistan has a crucial obligation to act progressively to overhaul and synchronize pharmacy education and practices. Not only do pharmacists need to work closely to deal with the continuing challenge of COVID-19, they do need to understand that their strength lies in the acquisition of identification rather than authority.

## Data Availability

Not applicable.
